# Evaluating the Hazard of Foetal Death following H1N1 Influenza Vaccination; A Population Based Cohort Study in the UK GPRD

**DOI:** 10.1371/journal.pone.0051734

**Published:** 2012-12-10

**Authors:** Cormac J. Sammon, Julia Snowball, Anita McGrogan, Corinne S. de Vries

**Affiliations:** Department of Pharmacy and Pharmacology, University of Bath, Bath, United Kingdom; Tehran University of Medical Sciences, Iran (Republic of Islamic)

## Abstract

**Background:**

To evaluate the risk of foetal loss associated with pandemic influenza vaccination in pregnancy. Retrospective cohort study. UK General Practice Research Database Pregnancies ending in delivery or spontaneous foetal death after 21 October 2009 and starting before 01 January 2010.

**Methodology/Principal Findings:**

Hazard ratios of foetal death for vaccinated compared to unvaccinated pregnancies were estimated for gestational weeks 9 to 12, 13 to 24 and 25 to 43 using discrete-time survival analysis. Separate models were specified to evaluate whether the potential effect of vaccination on foetal loss might be transient (for ∼4 weeks post vaccination only) or more permanent (for the duration of the pregnancy). 39,863 pregnancies meeting our inclusion criteria contributed a total of 969,322 gestational weeks during the study period. 9,445 of the women were vaccinated before or during pregnancy. When the potential effect of vaccination was assumed to be transient, the hazard of foetal death during gestational weeks 9 through 12 (HR_unadj_ 0.56; CI_95_ 0.43 to 0.73) and 13 through 24 (HR_unadj_ 0.45; CI_95_ 0.28 to 0.73) was lower in the 4 weeks after vaccination than in other weeks. Where the more permanent exposure definition was specified, vaccinated pregnancies also had a lower hazard of foetal loss than unvaccinated pregnancies in gestational weeks 9 through 12 (HR_unadj_ 0.74; CI_95_ 0.62 to 0.88) and 13 through 24 (HR_unadj_ 0.59; CI_95_ 0.45 to 0.77). There was no difference in the hazard of foetal loss during weeks 25 to 43 in either model. Sensitivity analyses suggest the strong protective associations observed may be due in part to unmeasured confounding.

**Conclusions/Significance:**

Influenza vaccination during pregnancy does not appear to increase the risk of foetal death. This study therefore supports the continued recommendation of influenza vaccination of pregnant women.

## Introduction

Current evidence suggests the risk/benefit profile of influenza vaccination in pregnancy is favourable for both the mother and her newborn. The benefits of vaccination to the mother are particularly evident in the second and third trimester and during pandemics [1], [2]. This is reflected in national immunisation policies implemented in countries throughout the world [3]. Despite this, little is known about the effects of influenza and influenza vaccination on the developing foetus. A small number of studies have linked influenza infection to an increased rate of foetal death [4], [5], [6], [7], babies born small for their gestational age [8] and prematurity [8]. If influenza infection does increase the risk of these adverse pregnancy outcomes, vaccination might prove beneficial in mitigating this risk. However, given the paucity of evidence available, few public health authorities currently cite influenza-related adverse pregnancy outcomes as their rationale for recommending influenza vaccination of pregnant women.

With regard to foetal risks, little is known about the potential adverse effect that influenza vaccination may have. While maternal safety can be extrapolated to a certain extent from the general population, it is not possible to extrapolate risks to the foetus from other populations. Given ethical issues concerning the inclusion of pregnant women in randomised controlled trials, most studies that have considered influenza vaccine safety in pregnancy have been observational in nature. Those that have evaluated the vaccine-associated risk of adverse pregnancy outcomes have focused on outcomes such as preterm birth [9], [10], malformations [10], [11], [12] and caesarean section [9]. Few studies have investigated the risk of pregnancy loss (miscarriages/stillbirths) following influenza vaccination [12] as there are a number of methodological challenges inherent in studying such associations. Bias introduced by the incomplete ascertainment of implantation failures and early embryonic deaths is the primary problem; ∼60% of conceptions are lost prior to clinical recognition [13], while variation in both exposure and outcome [14] incidence over gestational time may also create challenges. If these issues are not accounted for appropriately in study design and analysis they can result in profoundly biased risk estimates.

While influenza vaccination in pregnancy is recommended in the UK and many other countries, uptake of influenza vaccines by pregnant women is low [15], [16], [17], [18], [19], [20]. Perceptions that influenza infection is not dangerous, and vaccine safety concerns have been identified as major barriers to uptake of both pandemic [21], [22], [23], [24], [25] and seasonal [24], [26], [27] influenza vaccine in pregnant women. Without insight into the risk of pregnancy loss associated with vaccination it will be difficult to achieve a meaningful increase in vaccination uptake. In this study we have investigated whether the hazard of foetal death is altered in pregnancies vaccinated against influenza A(H1N1)pdm09.

## Methods

We designed a cohort study in which we used discrete-time survival analysis to compare the hazard of foetal death occurring after 8 weeks gestation between vaccinated and unvaccinated pregnant women. Using survival analysis allowed us to account for the changing incidence of pregnancy losses and pandemic vaccination with increasing gestational age, while using a discrete parameterization of time acknowledged potential uncertainties in estimated last menstrual period (LMP) dates. Delaying study entry until the 9th week of gestation means we focus solely on pregnancy losses occurring after 8 weeks and therefore exclude the selection bias that would be introduced by the incomplete ascertainment of embryonic deaths. However, this also means any risk estimates reported in the study are conditional on the pregnancy surviving through at least the first 8 weeks gestation.

This study was carried out using the UK General Practice Research Database (GPRD). The GPRD is a primary care database containing the anonymised records of ∼8.4% of the UK population [28]. Patient data routinely available in the database include demographic details, diagnoses and symptoms leading to hospital admissions, immunisations, pregnancies, laboratory tests, referrals to specialists, prescriptions issued by the GP, contraception, hospital discharge and clinic summaries and deaths [29]. The GPRD operates a continuous quality control procedure and check that all data submitted by practices meet a specific set of quality criteria; those meeting the criteria are considered of a standard sufficient for research purposes.

**Figure 1:**
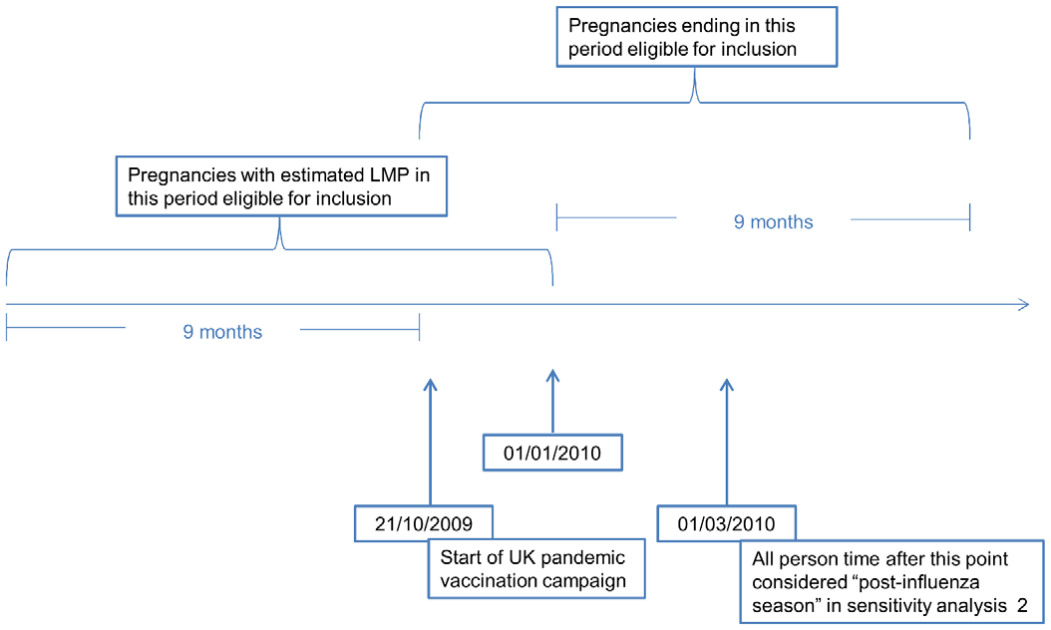
Time periods for inclusion of pregnancies in the study.

The study population consisted of all women with a pregnancy ending after the start of the vaccination campaign on 21 October 2009 and starting before 1 January 2010, for whom at least 6 months of data was available before their LMP date (Figure 1). Pregnancies were identified using an algorithm similar to those described elsewhere [30], [31]. In summary this algorithm identifies individual pregnancies based on records of pregnancy outcomes and estimates each pregnancy's start and end date using all pregnancy related events in a woman's record. Where a pregnancy outcome was identified but the pregnancy start date remained unclear the pregnancy was assigned a default start date of 280 days before the date of delivery/stillbirth or 70 days before the date of foetal death. This algorithmic approach to pregnancy identification on the GPRD has been verified using manual review of electronic and paper medical records [32]. Ectopic pregnancies and pregnancies resulting in hydatidiform moles or induced abortions were excluded from the study population and where a woman had multiple eligible pregnancies only the first pregnancy was included. Previous work suggested pandemic vaccinations may be misclassified in Northern Irish GPRD practices therefore pregnancies in women registered with Northern Irish practices were excluded.

The main outcome in this study was foetal death, defined as a pregnancy loss at any time between the 9^th^ gestational week and the onset of labour/delivery. Foetal death includes first trimester miscarriages (gestational weeks 9–12), second trimester miscarriages (gestational weeks 13–24) and second or third trimester stillbirths (gestational weeks 25+).

Pandemic influenza vaccinations were identified using influenza A(H1N1)pdm09 vaccine specific medical and product codes; codes which had been created to allow differential recording of seasonal and pandemic vaccinations on GP systems [33]. Where a woman had more than one pandemic vaccination recorded the first record of vaccination was considered to be on the vaccination date.

Potential confounders and effect modifiers identified *a priori* and investigated in the analysis include: maternal age, history of spontaneous loss, diabetes, pre-pregnancy smoking status, pre-pregnancy alcohol use, pre-pregnancy body mass index, the number of consultations in the 6 months before the LMP date and being in an influenza A(H1N1)pdm09 clinical risk group (i.e. recommended for pandemic influenza vaccination due to a chronic medical condition). A separate category was created for all those with missing data on pre-pregnancy smoking status, alcohol use or BMI.

In the discrete survival model, weekly intervals were used to define exposure and event occurrence and separate hazard ratios were estimated for weeks 9–12, weeks 13–24 and weeks 25–42. Delayed entry was used to account for left truncation of pregnancies beginning before the start of the study period. Influenza A(H1N1)pdm09 vaccination status was coded as a time varying covariate. We used two influenza A(H1N1)pdm09 vaccine exposure definitions to represent the two separate hypotheses under investigation:

- To assess whether vaccination might have an acute adverse effect on pregnancy outcome we assumed exposure to be transient and investigated whether there was an association between influenza A(H1N1)pdm09 vaccination and foetal death in the week of vaccination or the three weeks immediately thereafter.
- To assess whether immunisation might protect against foetal death by conferring immunity against influenza and its related morbidity, we assumed exposure to be permanent and investigated whether there was an association between influenza A(H1N1)pdm09 vaccination and foetal death in any subsequent week of pregnancy.

Henceforth these two models shall be referred to as the ‘toxicity model’ (a) and the ‘immunity model’ (b). Effect modification was identified through stratification and introduction of interaction terms into the models. Confounders were defined as variables whose inclusion in the model changed the point estimate of the HR for vaccination by >10%. The proportionality assumption was investigated within each gestational period under investigation through the introduction of interaction terms between each variable and gestational age. All statistical analyses were carried out using STATA 12.

Previous work identifying pregnancies on the GPRD suggested that it would not be possible to ascertain the exact pregnancy start date for a large proportion of the first trimester spontaneous losses. In the main analysis a default pregnancy start date of 70 days before foetal death was assigned to any such pregnancies; as a result in the main analysis all such foetal deaths were defined as occurring in the 10^th^ week. To investigate the sensitivity of our estimates to this defaulting of first trimester pregnancy losses we estimated models in which we changed the default pregnancy start date to 42, 56 and 84 days before loss, defining foetal deaths as occurring in the 6^th^, 8^th^ or 12^th^ week respectively.

Vaccination does not confer immediate immunity on an individual; there is approximately a 1–2 week delay between influenza vaccination and the onset of immunity. As a sensitivity analysis for the immunity model we therefore coded both one- and two-week periods after vaccination as unexposed with “exposure” only beginning after immunity could plausibly have developed.

In order to investigate whether the associations observed in the ‘immunity’ model were due to underlying differences between individuals who were vaccinated and those who were not (a “healthy user effect”) we performed an additional analysis stratifying gestational weeks as <1 March 2010 or >28 February 2010. As influenza was not circulating widely after February 2010 [34] little or no protective association should be observed in this period; any association that was observed could therefore be considered an estimate of the level of confounding present in our main model estimates.

A fourth sensitivity analysis modelled the effect of a hypothetical confounder on our results [35]. It investigated whether some unmeasured factor, such as a healthy lifestyle, might be both associated with a decreased risk of foetal death and more prevalent among vaccinated than unvaccinated pregnancies.

## Results

**Figure 2:**
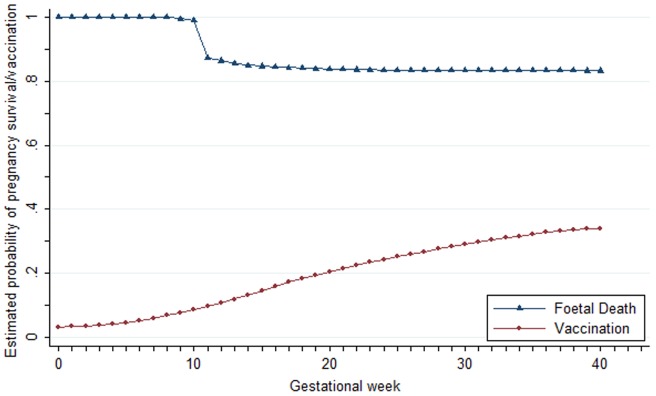
Percentage of pregnancies surviving (blue) and vaccinated (red) by each gestational week. The drop in survival at 10 weeks is an artefact of the defaulting process. In reality the losses contributing to this curve would be more evenly distributed across weeks 9–12 resulting in a more gradual drop in survival.

39,863 pregnancies meeting our inclusion criteria contributed a total of 969,322 gestational weeks during the study period. 36,438 of these pregnancies ended in a delivery and 3,425 ended in foetal death. 9,445 of the women had been immunised with an influenza vaccination before the end of their pregnancy and 9,161 of the vaccinations occurred during pregnancy. Patient characteristics are given in Table 1. The proportion of pregnancies vaccinated and the proportion of foetal deaths occurring over gestational time are shown in Figure 2.

**Table 1:** Population characteristics of pregnant women eligible for influenza vaccination during the influenza A(H1N1)pdm09 pandemic.

	Delivery		Foetal death					
			Week 9–12		Week 13–24		Week 25–41	
	n	%	n	%	n	%	n	%
**Total**	36,438	91.4	2,543	6.4	711	1.9	171	0.4
**Mean pregnancy length, weeks ** ***(SD)***	40.8	*−1.4*	10.2	*(0.6)*	16.6	*(3.5)*	36	*(6)*
**LMP date defaulted**								
** Yes**	19,902	54.6	2,026	79.7	21	3	90	52.6
** No**	16,536	45.4	517	20.3	690	97	81	47.4
**Unvaccinated weeks**								
** weeks 1–12**	59,425	9	4,912	100	2,081	44.3	298	10.3
** weeks 13–24**	215,195	32.5	-	-	2,614	55.7	1,182	40.8
** weeks 25–43**	388,192	58.6	-	-	-	-	1,416	48.9
**Vaccinated (weeks)**								
** weeks 1–12**	4,897	2.9	269	100	142	35.9	12	2.7
** weeks 13–24**	41,554	24.4	-		253	64.1	141	32
** weeks 25–43**	123,658	72.7	-		-	-	288	65.3
**Maternal age (years)**								
** Mean ** ***(SD)***	29.9	*(6)*	32.2	*(7.2)*	31.6	*(6.7)*	30	*(6.4)*
** Nov–19**	1,194	3.3	93	3.7	21	3	8	4.7
** 20–34**	25,350	69.6	1,371	53.9	422	59.4	117	68.4
** 35–40**	7,594	20.8	619	24.3	156	21.9	33	19.3
** 40–44**	2,151	5.9	393	15.5	102	14.3	12	7
** 45–49**	149	0.4	67	2.6	10	1.4	1	0.6
**Number of previous spontaneous abortions**								
** 0**	30,089	82.6	376	14.8	522	73.4	136	79.5
** 1**	5,175	14.2	1,624	63.9	160	22.5	25	14.6
** 2**	929	2.5	379	14.9	22	3.1	7	4.1
** >2**	245	0.7	164	6.4	7	1	3	1.8
**In clinical risk group for influenza vaccination**								
** No**	34,304	94.1	2,359	92.8	669	94.1	164	95.9
** Yes**	2,134	5.9	184	7.2	42	5.9	7	4.1
**Diabetes**								
** No**	36,136	99.2	2,501	98.3	700	98.5	168	98.2
** Yes**	302	0.8	42	1.7	11	1.5	3	1.8
**Number of consultations in 6 months before LMP**								
** 0–1**	8,664	23.8	554	21.8	158	22.2	47	27.5
** 02–Mar**	8,199	22.5	534	21	177	24.9	43	25.1
** 04–May**	6,095	16.7	404	15.9	101	14.2	28	16.4
** 06–Sep**	7,258	19.9	506	19.9	133	18.7	36	21.1
** 10+**	6,222	17.1	545	21.4	142	20	17	9.9
**Pre-pregnancy smoking status**								
** Smoker**	8,973	24.6	658	25.9	185	26	53	31
** Non-smoker**	19,751	54.2	1,327	52.2	383	53.9	82	48
** Ex-smoker**	7,491	20.6	534	21	141	19.8	36	21.1
** Unknown**	223	0.6	24	0.9	2	0.3	0	0
**Pre-pregnancy BMI**								
** <20**	3,727	10.2	247	9.7	66	9.3	21	12.3
** 20–24**	13,660	37.5	922	36.3	255	35.9	49	28.7
** 25–29**	7,157	19.6	498	19.6	167	23.5	37	21.6
** 30–34**	2,642	7.3	185	7.3	63	8.9	13	7.6
** >34**	2,084	5.7	175	6.9	45	6.3	16	9.4
** Unknown**	7168	19.7	516	20.3	115	16.2	35	20.5
**Pre-pregnancy alcohol consumption**								
** Drinker**	21,806	59.8	434	17.1	434	61.0	94	55.0
** Non-drinker**	7,850	21.5	168	6.6	168	23.6	38	22.2
** Heavy drinker**	354	1.0	9	0.4	9	1.3	3	1.8
** Unknown**	6428	17.6	100	3.9	100	14.1	36	21.1

The results of the main analyses are shown in Table 2. Both in the toxicity model and in the immunity model, the hazard of foetal death was reduced after A/H1N1pdm09 vaccinations in each of weeks 9 to 24 of gestation. This association appeared to be strongest in the toxicity model. After gestational week 24, no statistically significant associations were observed.

**Table 2:** Hazard ratios and 95% confidence intervals for association between pandemic influenza vaccination and foetal death in different gestational periods.

	“Immunity model”		“Toxicity model”	
	HR _unadj_	CI_95_	HR _unadj_	CI_95_
Foetal death in weeks 9–12	0.74	(0.62–0.88)	0.56	(0.43–0.73)
Foetal death in weeks 13–24	0.59	(0.45–0.77)	0.45	(0.28–0.73)
Foetal death in weeks 25–43	0.70	(0.47–1.03)	1.56	(0.73–3.34)

As anticipated, maternal age, number of previous spontaneous losses, being in a influenza clinical risk group and having diabetes were all associated with the hazard of foetal death. However, no variables were observed to confound the association between vaccination and foetal death. Hazard ratios and confidence intervals for the missing categories did not suggest they masked a confounding association (data not shown and available on request). Fitting interactions between the vaccination and gestational age suggested that the hazards across vaccination groups were proportional within each gestational period reported (data not shown and available on request).

For 2,025 of 2,543 first trimester foetal deaths, no information was available regarding the LMP date or the expected date of delivery. These pregnancies were assigned a default pregnancy start date of 63 to 70 days before the date of loss (i.e. foetal death occurred in the 10^th^ gestational week). In a sensitivity analysis, as we moved the default start date forward, and therefore decreased the estimated gestational age of many of the first trimester foetal deaths, our risk estimates moved closer to unity (Table 3).

**Table 3:** Sensitivity analysis 1.

	“Immunity model”		“Toxicity model”	
	HR_unadj_	CI_95_	HR_unadj_	CI_95_
default 6th week	1.24	(1.04–1.48)	1.06	(0.82–1.38)
default 8th week	0.98	(0.83–1.17)	0.78	(0.60–1.00)
default 10th week**	0.74	(0.62–0.88)	0.56	(0.43–0.73)
default 12th week	0.59	(0.49–0.70)	0.44	(0.35–0.58)

All hazard ratios are for gestational weeks 9–12 only. **same as effect estimates in table 2.

Effect of varying the default length of first trimester spontaneous losses.

When we changed exposure status in the first two weeks following vaccination to unexposed (to allow for delay between vaccination and onset of immunity) we observed a decrease in the protective association observed. This was most notable in the 9–12 week gestational period when the rates of vaccination and loss were changing rapidly (Table 4). The protective association with influenza A(H1N1)pdm09 vaccine was of a similar magnitude during periods of high influenza circulation and during periods of little/no influenza circulation (Table 5).

**Table 4:** Sensitivity analysis 2.

		“Immunity model”	
		HR_unadj_	CI_95_
**week of vaccination** coded as unexposed	loss in weeks 9–12	0.80	(0.66–0.96)
	loss in weeks 13–24	0.63	(0.48–0.83)
	loss in weeks 25–43	0.69	(0.47–1.02)
week of vaccination **and week following vaccination** coded as unexposed	loss in weeks 9–12	0.84	(0.69–1.03)
	loss in weeks 13–24	0.64	(0.48–0.85)
	loss in weeks 25–43	0.69	(0.46–1.02)

One and two week post vaccination time periods coded as unexposed to account for a delay between vaccination and onset of immunity.

**Table 5:** Sensitivity analysis 3.

		“Immunity model”		
		*HR_unadj_		CI_95_
Influenza season	loss in weeks 9–12	0.76		(0.63–0.92)
	loss in weeks 13–24	0.55		(0.40–0.75)
			
	loss in weeks 25–43	0.70		(0.38–1.29)
Post-influenza season	loss in weeks 9–12	0.63		(0.39–1.05)
	loss in weeks 13–24	0.68		(0.42–1.10)
	loss in weeks 25–43		0.71	(0.43–1.18)

Pregnancy weeks stratified as being either during influenza season or post-influenza season; no causal protective associations are expected in the post-influenza season period.

The sensitivity analysis modelling the effect of a hypothetical confounder suggested that in order for a confounder to completely account for the protective associations observed in the immunity model or to mask an adverse association in the toxicity model it would have to be both considerably more prevalent among the vaccinated than unvaccinated and strongly associated with a decreased risk of foetal death (Table S1). Taking healthy lifestyle as an example, if 90% of vaccinated women followed this healthy lifestyle and only 20% of unvaccinated women did, the healthy lifestyle factor would have to be associated with a 40% reduced risk of foetal death to produce the protective associations observed in weeks 9–12 or a 50% reduced risk to produce the protective association in weeks 13–24. A similarly distributed healthy lifestyle would have to be associated with a reduction in the risk of foetal death of 70%–80% to hide an acute adverse effect in weeks 9–12 or 13–24.

## Discussion

Vaccination against influenza A(H1N1)pdm09 was associated with a lower risk of foetal death. While this may be explained in part or completely by residual uncontrolled confounding, this study provides reassurance that vaccination is unlikely to be associated with an increased risk of pregnancy loss.

To our knowledge this is one of the first large population based studies of the association between influenza A(H1N1)pdm09 vaccination and foetal death [36], [37]. As the influenza A(H1N1)pdm09 vaccine most commonly used in the UK was the AS03 adjuvanted vaccine, Pandemrix^®^, this is also one of the first studies to investigate the association between an adjuvanted vaccine and foetal loss. The rates of foetal death and vaccine uptake observed in the GPRD are in line with rates observed elsewhere [16], [38], [39]. As the A(H1N1)pdm09 vaccine was primarily administered in GP surgeries, the accuracy of vaccination information in the GPRD should be high. However, misclassification of vaccination status may have occurred where pandemic vaccinations were recorded using non specific influenza vaccination codes or obtained from outside the GP practice. The use of a discrete-time survival analysis enabled us to account for the opposing trends in the incidence of vaccine uptake and foetal death during pregnancy, while acknowledging uncertainties in the estimated gestational age of event occurrence. We were able to examine a number of potential confounders in this study; however sensitivity analyses suggested residual confounding, for instance by lifestyle or dietary factors or folic acid intake, remained present.

The observation of a protective association immediately after vaccination and during periods of little/no influenza circulation is suggestive of unmeasured confounding as the vaccine can provide little or no true protective effect in these periods. There is a possibility that women may begin to feel ill or be admitted to hospital in the days preceding foetal death, if this were the case they would be unlikely to be vaccinated in such a period. This could explain the protective associations observed in both models and the stronger association observed in the toxicity model. The weakening of the protective associations with changing the exposure definition may result from the partial removal of such bias (Table 4). While the potential influence of residual confounding on our estimates needs to be carefully considered, it is reassuring that the risk estimates were reasonably precise and no statistically significant increases in the hazard of foetal death were observed in any of the sensitivity analyses evaluating this.

The sharp drop in survival at 10 weeks in Figure 2 results from the defaulting of LMP dates of first trimester foetal deaths to 10 weeks before the date of loss; the results were sensitive to misclassification resulting from such defaulting. However, sensitivity analysis results suggest it is unlikely that any such misclassification would be substantial enough to hide an increased hazard of foetal death among vaccinated pregnancies (Table 3) as a significantly increased hazard was only observed when the default LMP date of first trimester foetal deaths was set to 6 weeks before the foetal death; an unlikely situation. In view of missing information on early pregnancy loss on the GPRD we excluded pregnancy losses occurring before 9 weeks gestation from our analysis; this study therefore provides no insight into the risk of embryonic death following vaccination. However, most pregnant women do not contact their GP for their pregnancy until close to the end of the embryonic period; exposure to influenza vaccine is therefore low, and mainly limited to those in clinical risk groups, early in pregnancy.

A number of recent studies have investigated the risk of foetal death following H1N1 vaccination. Pasternak *et al* reported non- or marginally-significant differences in the propensity score adjusted hazard of overall foetal death (HR 0.79; CI_95_ 0.53 to 1.16), spontaneous loss (HR 1.11; CI_95_ 0.71 to 1.73) and stillbirth (HR 0.44; CI_95_ 0.20 to 0.94) between vaccinated and unvaccinated women; these point estimates and those from their sensitivity analyses generally suggest a lower hazard of foetal death among vaccinated women [36]. Recently, Fell *et al.* evaluated the risk of a range of pregnancy outcomes following influenza A(H1N1)pdm09 vaccination, reporting an adjusted RR of foetal death after 20 weeks of 0.66 (CI_95_ 0.47 to 0.91)[37]. Restricting our analysis to foetal deaths occurring after 20 weeks for comparison, we observed a HR of a similar magnitude (HR 0.62; CI_95_ 0.46 to 0.84). In a primarily methodological paper, Xu *et al* compared the rate of spontaneous loss in H1N1 vaccinated women contacting North American teratology information services to that in unvaccinated women contacting the same service [40]. While study power was low, spontaneous loss rates among the vaccinated were similar to those in the unvaccinated. Tavares *et al* and Moro *et al* have reported rates of spontaneous loss among vaccinated pregnant women with both finding rates to be within the range expected [41], [42].

Reassuringly, while the protective associations reported in some of these studies may be completely explained by underlying differences between women who choose to be vaccinated and those who do not, none found any evidence to suggest an increase in the risk of foetal death following vaccination. Indeed, sensitivity analyses suggest that a confounder would have to be both strongly protective against foetal death and highly prevalent among vaccinated women for it to hide an adverse association between vaccination and foetal death.

As methodological difficulties and low exposure prevalence complicate the evaluation of the risk of embryonic death, future study may be better directed at further evaluating the risk of adverse pregnancy outcomes such as foetal death, malformations, preterm birth and growth retardation. Developing methods to account for, or evaluate, residual confounding will be vital in any such studies. While this study does not provide any definitive evidence that influenza vaccination in pregnancy is completely safe or effective, its results provide some reassurance to patients that vaccination is unlikely to increase the risk of foetal death. Taken alongside current evidence, this study supports a favourable risk-benefit profile of influenza vaccines and the continued recommendation of influenza vaccination of pregnant women.

## Supporting Information

Table S1 **Sensitivity analysis 4.** Modelling the effect of a hypothetical confounder on the hazard of foetal death.
(DOCX)

Table S2 **Patient characteristics among pregnancies with and without data recorded on LMP date.**
(DOCX)
