# Supplementary material for: Evaluating the Hazard of Foetal Death following H1N1 Influenza Vaccination; A Population Based Cohort Study in the UK GPRD
Source: PLoS One. 2012 Dec 10;7(12):e51734. doi: 10.1371/journal.pone.0051734 (PMC3541601; doi:10.1371/journal.pone.0051734)
Supplement: Table S1 — Sensitivity analysis 4. Modelling the effect of a hypothetical confounder on the hazard of foetal death. (DOCX) [file pone.0051734.s001.docx]

| **Notation** | | |
| --- | --- | --- |
|  | |  |
| RR | “True” or fully adjusted exposure relative risk | |
| ARR | Apparent (or observed) exposure relative risk | |
| RR_CD_ | Association between confounder and disease outcome | |
| P_C1_ | Prevalence of confounder in the exposed | |
| P_C0_ | Prevalence of confounder in the unexposed | |

Table S1.1 The effect of a hypothetical confounder of varying strength and prevalence on the hazard ratio of foetal death in gestational weeks 9-12 (immunity model)

| fix | X | Y | fix | Z_2_ | Z_1_ |
| --- | --- | --- | --- | --- | --- |
| **ARR** | **RR_CD_** | **P_C1_** | **P_C0_** | **RR_adjusted_** | **% Bias** |
| 0.74 | 0.5 | 0.6 | 0.4 | 0.85 | -12.50 |
| 0.74 | 0.6 | 0.6 | 0.4 | 0.82 | -9.52 |
| 0.74 | 0.7 | 0.6 | 0.4 | 0.79 | -6.82 |
| 0.74 | 0.8 | 0.6 | 0.4 | 0.77 | -4.35 |
| 0.74 | 0.9 | 0.6 | 0.4 | 0.76 | -2.08 |
| 0.74 | 0.5 | 0.7 | 0.4 | 0.91 | -18.75 |
| 0.74 | 0.6 | 0.7 | 0.4 | 0.86 | -14.29 |
| 0.74 | 0.7 | 0.7 | 0.4 | 0.82 | -10.23 |
| 0.74 | 0.8 | 0.7 | 0.4 | 0.79 | -6.52 |
| 0.74 | 0.9 | 0.7 | 0.4 | 0.76 | -3.12 |
| 0.74 | 0.5 | 0.8 | 0.4 | 0.99 | -25.00 |
| 0.74 | 0.6 | 0.8 | 0.4 | 0.91 | -19.05 |
| 0.74 | 0.7 | 0.8 | 0.4 | 0.86 | -13.64 |
| 0.74 | 0.8 | 0.8 | 0.4 | 0.81 | -8.70 |
| 0.74 | 0.9 | 0.8 | 0.4 | 0.77 | -4.17 |
| 0.74 | 0.5 | 0.9 | 0.4 | 1.08 | -31.25 |
| 0.74 | 0.6 | 0.9 | 0.4 | 0.97 | -23.81 |
| 0.74 | 0.7 | 0.9 | 0.4 | 0.89 | -17.05 |
| 0.74 | 0.8 | 0.9 | 0.4 | 0.83 | -10.87 |
| 0.74 | 0.9 | 0.9 | 0.4 | 0.78 | -5.21 |
| 0.74 | 0.5 | 0.6 | 0.4 | 0.85 | -12.50 |
| 0.74 | 0.6 | 0.6 | 0.3 | 0.86 | -13.64 |
| 0.74 | 0.7 | 0.6 | 0.3 | 0.82 | -9.89 |
| 0.74 | 0.8 | 0.6 | 0.3 | 0.79 | -6.38 |
| 0.74 | 0.9 | 0.6 | 0.3 | 0.76 | -3.09 |
| 0.74 | 0.5 | 0.7 | 0.3 | 0.97 | -23.53 |
| 0.74 | 0.6 | 0.7 | 0.3 | 0.90 | -18.18 |
| 0.74 | 0.7 | 0.7 | 0.3 | 0.85 | -13.19 |
| 0.74 | 0.8 | 0.7 | 0.3 | 0.81 | -8.51 |
| 0.74 | 0.9 | 0.7 | 0.3 | 0.77 | -4.12 |
| 0.74 | 0.5 | 0.8 | 0.3 | 1.05 | -29.41 |
| 0.74 | 0.6 | 0.8 | 0.3 | 0.96 | -22.73 |
| 0.74 | 0.7 | 0.8 | 0.3 | 0.89 | -16.48 |
| 0.74 | 0.8 | 0.8 | 0.3 | 0.83 | -10.64 |
| 0.74 | 0.9 | 0.8 | 0.3 | 0.78 | -5.15 |
| 0.74 | 0.5 | 0.9 | 0.3 | 1.14 | -35.29 |
| 0.74 | 0.6 | 0.9 | 0.3 | 1.02 | -27.27 |
| 0.74 | 0.7 | 0.9 | 0.3 | 0.92 | -19.78 |
| 0.74 | 0.8 | 0.9 | 0.3 | 0.85 | -12.77 |
| 0.74 | 0.9 | 0.9 | 0.3 | 0.79 | -6.19 |
| 0.74 | 0.5 | 0.6 | 0.3 | 0.90 | -17.65 |
| 0.74 | 0.6 | 0.6 | 0.2 | 0.90 | -17.39 |
| 0.74 | 0.7 | 0.6 | 0.2 | 0.85 | -12.77 |
| 0.74 | 0.8 | 0.6 | 0.2 | 0.81 | -8.33 |
| 0.74 | 0.9 | 0.6 | 0.2 | 0.77 | -4.08 |
| 0.74 | 0.5 | 0.7 | 0.2 | 1.02 | -27.78 |
| 0.74 | 0.6 | 0.7 | 0.2 | 0.95 | -21.74 |
| 0.74 | 0.7 | 0.7 | 0.2 | 0.88 | -15.96 |
| 0.74 | 0.8 | 0.7 | 0.2 | 0.83 | -10.42 |
| 0.74 | 0.9 | 0.7 | 0.2 | 0.78 | -5.10 |
| 0.74 | 0.5 | 0.8 | 0.2 | 1.11 | -33.33 |
| 0.74 | 0.6 | 0.8 | 0.2 | 1.00 | -26.09 |
| 0.74 | 0.7 | 0.8 | 0.2 | 0.92 | -19.15 |
| 0.74 | 0.8 | 0.8 | 0.2 | 0.85 | -12.50 |
| 0.74 | 0.9 | 0.8 | 0.2 | 0.79 | -6.12 |
| 0.74 | 0.5 | 0.9 | 0.2 | 1.21 | -38.89 |
| 0.74 | 0.6 | 0.9 | 0.2 | 1.06 | -30.43 |
| 0.74 | 0.7 | 0.9 | 0.2 | 0.95 | -22.34 |
| 0.74 | 0.8 | 0.9 | 0.2 | 0.87 | -14.58 |
| 0.74 | 0.9 | 0.9 | 0.2 | 0.80 | -7.14 |

Table S1.2 The effect of a hypothetical confounder of varying strength and prevalence on the hazard ratio of foetal death in gestational weeks 13-24 (immunity model)

| fix | X | Y | fix | Z_2_ | Z_1_ |
| --- | --- | --- | --- | --- | --- |
| **ARR** | **RR_CD_** | **P_C1_** | **P_C0_** | **RR_adjusted_** | **% Bias** |
| 0.59 | 0.5 | 0.6 | 0.4 | 0.67 | -12.50 |
| 0.59 | 0.6 | 0.6 | 0.4 | 0.65 | -9.52 |
| 0.59 | 0.7 | 0.6 | 0.4 | 0.63 | -6.82 |
| 0.59 | 0.8 | 0.6 | 0.4 | 0.62 | -4.35 |
| 0.59 | 0.9 | 0.6 | 0.4 | 0.60 | -2.08 |
| 0.59 | 0.5 | 0.7 | 0.4 | 0.73 | -18.75 |
| 0.59 | 0.6 | 0.7 | 0.4 | 0.69 | -14.29 |
| 0.59 | 0.7 | 0.7 | 0.4 | 0.66 | -10.23 |
| 0.59 | 0.8 | 0.7 | 0.4 | 0.63 | -6.52 |
| 0.59 | 0.9 | 0.7 | 0.4 | 0.61 | -3.12 |
| 0.59 | 0.5 | 0.8 | 0.4 | 0.79 | -25.00 |
| 0.59 | 0.6 | 0.8 | 0.4 | 0.73 | -19.05 |
| 0.59 | 0.7 | 0.8 | 0.4 | 0.68 | -13.64 |
| 0.59 | 0.8 | 0.8 | 0.4 | 0.65 | -8.70 |
| 0.59 | 0.9 | 0.8 | 0.4 | 0.62 | -4.17 |
| 0.59 | 0.5 | 0.9 | 0.4 | 0.86 | -31.25 |
| 0.59 | 0.6 | 0.9 | 0.4 | 0.77 | -23.81 |
| 0.59 | 0.7 | 0.9 | 0.4 | 0.71 | -17.05 |
| 0.59 | 0.8 | 0.9 | 0.4 | 0.66 | -10.87 |
| 0.59 | 0.9 | 0.9 | 0.4 | 0.62 | -5.21 |
| 0.59 | 0.5 | 0.6 | 0.4 | 0.67 | -12.50 |
| 0.59 | 0.6 | 0.6 | 0.3 | 0.68 | -13.64 |
| 0.59 | 0.7 | 0.6 | 0.3 | 0.65 | -9.89 |
| 0.59 | 0.8 | 0.6 | 0.3 | 0.63 | -6.38 |
| 0.59 | 0.9 | 0.6 | 0.3 | 0.61 | -3.09 |
| 0.59 | 0.5 | 0.7 | 0.3 | 0.77 | -23.53 |
| 0.59 | 0.6 | 0.7 | 0.3 | 0.72 | -18.18 |
| 0.59 | 0.7 | 0.7 | 0.3 | 0.68 | -13.19 |
| 0.59 | 0.8 | 0.7 | 0.3 | 0.64 | -8.51 |
| 0.59 | 0.9 | 0.7 | 0.3 | 0.62 | -4.12 |
| 0.59 | 0.5 | 0.8 | 0.3 | 0.84 | -29.41 |
| 0.59 | 0.6 | 0.8 | 0.3 | 0.76 | -22.73 |
| 0.59 | 0.7 | 0.8 | 0.3 | 0.71 | -16.48 |
| 0.59 | 0.8 | 0.8 | 0.3 | 0.66 | -10.64 |
| 0.59 | 0.9 | 0.8 | 0.3 | 0.62 | -5.15 |
| 0.59 | 0.5 | 0.9 | 0.3 | 0.91 | -35.29 |
| 0.59 | 0.6 | 0.9 | 0.3 | 0.81 | -27.27 |
| 0.59 | 0.7 | 0.9 | 0.3 | 0.74 | -19.78 |
| 0.59 | 0.8 | 0.9 | 0.3 | 0.68 | -12.77 |
| 0.59 | 0.9 | 0.9 | 0.3 | 0.63 | -6.19 |
| 0.59 | 0.5 | 0.6 | 0.3 | 0.72 | -17.65 |
| 0.59 | 0.6 | 0.6 | 0.2 | 0.71 | -17.39 |
| 0.59 | 0.7 | 0.6 | 0.2 | 0.68 | -12.77 |
| 0.59 | 0.8 | 0.6 | 0.2 | 0.64 | -8.33 |
| 0.59 | 0.9 | 0.6 | 0.2 | 0.62 | -4.08 |
| 0.59 | 0.5 | 0.7 | 0.2 | 0.82 | -27.78 |
| 0.59 | 0.6 | 0.7 | 0.2 | 0.75 | -21.74 |
| 0.59 | 0.7 | 0.7 | 0.2 | 0.70 | -15.96 |
| 0.59 | 0.8 | 0.7 | 0.2 | 0.66 | -10.42 |
| 0.59 | 0.9 | 0.7 | 0.2 | 0.62 | -5.10 |
| 0.59 | 0.5 | 0.8 | 0.2 | 0.89 | -33.33 |
| 0.59 | 0.6 | 0.8 | 0.2 | 0.80 | -26.09 |
| 0.59 | 0.7 | 0.8 | 0.2 | 0.73 | -19.15 |
| 0.59 | 0.8 | 0.8 | 0.2 | 0.67 | -12.50 |
| 0.59 | 0.9 | 0.8 | 0.2 | 0.63 | -6.12 |
| 0.59 | 0.5 | 0.9 | 0.2 | 0.97 | -38.89 |
| 0.59 | 0.6 | 0.9 | 0.2 | 0.85 | -30.43 |
| 0.59 | 0.7 | 0.9 | 0.2 | 0.76 | -22.34 |
| 0.59 | 0.8 | 0.9 | 0.2 | 0.69 | -14.58 |
| 0.59 | 0.9 | 0.9 | 0.2 | 0.64 | -7.14 |

Table S1.3 The effect of a hypothetical confounder of varying strength and prevalence on the hazard ratio of foetal death in gestational weeks 9-12 (toxicity model)

| fix | X | Y | fix | Z_2_ | Z_1_ |
| --- | --- | --- | --- | --- | --- |
| **ARR** | **RR_CD_** | **P_C1_** | **P_C0_** | **RR_adjusted_** | **% Bias** |
| 0.56 | 0.5 | 0.6 | 0.4 | 0.64 | -12.50 |
| 0.56 | 0.6 | 0.6 | 0.4 | 0.62 | -9.52 |
| 0.56 | 0.7 | 0.6 | 0.4 | 0.60 | -6.82 |
| 0.56 | 0.8 | 0.6 | 0.4 | 0.59 | -4.35 |
| 0.56 | 0.9 | 0.6 | 0.4 | 0.57 | -2.08 |
| 0.56 | 0.5 | 0.7 | 0.4 | 0.69 | -18.75 |
| 0.56 | 0.6 | 0.7 | 0.4 | 0.65 | -14.29 |
| 0.56 | 0.7 | 0.7 | 0.4 | 0.62 | -10.23 |
| 0.56 | 0.8 | 0.7 | 0.4 | 0.60 | -6.52 |
| 0.56 | 0.9 | 0.7 | 0.4 | 0.58 | -3.12 |
| 0.56 | 0.5 | 0.8 | 0.4 | 0.75 | -25.00 |
| 0.56 | 0.6 | 0.8 | 0.4 | 0.69 | -19.05 |
| 0.56 | 0.7 | 0.8 | 0.4 | 0.65 | -13.64 |
| 0.56 | 0.8 | 0.8 | 0.4 | 0.61 | -8.70 |
| 0.56 | 0.9 | 0.8 | 0.4 | 0.58 | -4.17 |
| 0.56 | 0.5 | 0.9 | 0.4 | 0.81 | -31.25 |
| 0.56 | 0.6 | 0.9 | 0.4 | 0.74 | -23.81 |
| 0.56 | 0.7 | 0.9 | 0.4 | 0.68 | -17.05 |
| 0.56 | 0.8 | 0.9 | 0.4 | 0.63 | -10.87 |
| 0.56 | 0.9 | 0.9 | 0.4 | 0.59 | -5.21 |
| 0.56 | 0.5 | 0.6 | 0.4 | 0.64 | -12.50 |
| 0.56 | 0.6 | 0.6 | 0.3 | 0.65 | -13.64 |
| 0.56 | 0.7 | 0.6 | 0.3 | 0.62 | -9.89 |
| 0.56 | 0.8 | 0.6 | 0.3 | 0.60 | -6.38 |
| 0.56 | 0.9 | 0.6 | 0.3 | 0.58 | -3.09 |
| 0.56 | 0.5 | 0.7 | 0.3 | 0.73 | -23.53 |
| 0.56 | 0.6 | 0.7 | 0.3 | 0.68 | -18.18 |
| 0.56 | 0.7 | 0.7 | 0.3 | 0.65 | -13.19 |
| 0.56 | 0.8 | 0.7 | 0.3 | 0.61 | -8.51 |
| 0.56 | 0.9 | 0.7 | 0.3 | 0.58 | -4.12 |
| 0.56 | 0.5 | 0.8 | 0.3 | 0.79 | -29.41 |
| 0.56 | 0.6 | 0.8 | 0.3 | 0.72 | -22.73 |
| 0.56 | 0.7 | 0.8 | 0.3 | 0.67 | -16.48 |
| 0.56 | 0.8 | 0.8 | 0.3 | 0.63 | -10.64 |
| 0.56 | 0.9 | 0.8 | 0.3 | 0.59 | -5.15 |
| 0.56 | 0.5 | 0.9 | 0.3 | 0.87 | -35.29 |
| 0.56 | 0.6 | 0.9 | 0.3 | 0.77 | -27.27 |
| 0.56 | 0.7 | 0.9 | 0.3 | 0.70 | -19.78 |
| 0.56 | 0.8 | 0.9 | 0.3 | 0.64 | -12.77 |
| 0.56 | 0.9 | 0.9 | 0.3 | 0.60 | -6.19 |
| 0.56 | 0.5 | 0.6 | 0.3 | 0.68 | -17.65 |
| 0.56 | 0.6 | 0.6 | 0.2 | 0.68 | -17.39 |
| 0.56 | 0.7 | 0.6 | 0.2 | 0.64 | -12.77 |
| 0.56 | 0.8 | 0.6 | 0.2 | 0.61 | -8.33 |
| 0.56 | 0.9 | 0.6 | 0.2 | 0.58 | -4.08 |
| 0.56 | 0.5 | 0.7 | 0.2 | 0.78 | -27.78 |
| 0.56 | 0.6 | 0.7 | 0.2 | 0.72 | -21.74 |
| 0.56 | 0.7 | 0.7 | 0.2 | 0.67 | -15.96 |
| 0.56 | 0.8 | 0.7 | 0.2 | 0.63 | -10.42 |
| 0.56 | 0.9 | 0.7 | 0.2 | 0.59 | -5.10 |
| 0.56 | 0.5 | 0.8 | 0.2 | 0.84 | -33.33 |
| 0.56 | 0.6 | 0.8 | 0.2 | 0.76 | -26.09 |
| 0.56 | 0.7 | 0.8 | 0.2 | 0.69 | -19.15 |
| 0.56 | 0.8 | 0.8 | 0.2 | 0.64 | -12.50 |
| 0.56 | 0.9 | 0.8 | 0.2 | 0.60 | -6.12 |
| 0.56 | 0.5 | 0.9 | 0.2 | 0.92 | -38.89 |
| 0.56 | 0.6 | 0.9 | 0.2 | 0.81 | -30.43 |
| 0.56 | 0.7 | 0.9 | 0.2 | 0.72 | -22.34 |
| 0.56 | 0.8 | 0.9 | 0.2 | 0.66 | -14.58 |
| 0.56 | 0.9 | 0.9 | 0.2 | 0.60 | -7.14 |

Table S1.1 The effect of a hypothetical confounder of varying strength and prevalence on the hazard ratio of foetal death in gestational weeks 13-24 (toxicity model)

| fix | X | Y | fix | Z_2_ | Z_1_ |
| --- | --- | --- | --- | --- | --- |
| **ARR** | **RR_CD_** | **P_C1_** | **P_C0_** | **RR_adjusted_** | **% Bias** |
| 0.45 | 0.5 | 0.6 | 0.4 | 0.51 | -12.50 |
| 0.45 | 0.6 | 0.6 | 0.4 | 0.50 | -9.52 |
| 0.45 | 0.7 | 0.6 | 0.4 | 0.48 | -6.82 |
| 0.45 | 0.8 | 0.6 | 0.4 | 0.47 | -4.35 |
| 0.45 | 0.9 | 0.6 | 0.4 | 0.46 | -2.08 |
| 0.45 | 0.5 | 0.7 | 0.4 | 0.55 | -18.75 |
| 0.45 | 0.6 | 0.7 | 0.4 | 0.53 | -14.29 |
| 0.45 | 0.7 | 0.7 | 0.4 | 0.50 | -10.23 |
| 0.45 | 0.8 | 0.7 | 0.4 | 0.48 | -6.52 |
| 0.45 | 0.9 | 0.7 | 0.4 | 0.46 | -3.12 |
| 0.45 | 0.5 | 0.8 | 0.4 | 0.60 | -25.00 |
| 0.45 | 0.6 | 0.8 | 0.4 | 0.56 | -19.05 |
| 0.45 | 0.7 | 0.8 | 0.4 | 0.52 | -13.64 |
| 0.45 | 0.8 | 0.8 | 0.4 | 0.49 | -8.70 |
| 0.45 | 0.9 | 0.8 | 0.4 | 0.47 | -4.17 |
| 0.45 | 0.5 | 0.9 | 0.4 | 0.65 | -31.25 |
| 0.45 | 0.6 | 0.9 | 0.4 | 0.59 | -23.81 |
| 0.45 | 0.7 | 0.9 | 0.4 | 0.54 | -17.05 |
| 0.45 | 0.8 | 0.9 | 0.4 | 0.50 | -10.87 |
| 0.45 | 0.9 | 0.9 | 0.4 | 0.47 | -5.21 |
| 0.45 | 0.5 | 0.6 | 0.4 | 0.51 | -12.50 |
| 0.45 | 0.6 | 0.6 | 0.3 | 0.52 | -13.64 |
| 0.45 | 0.7 | 0.6 | 0.3 | 0.50 | -9.89 |
| 0.45 | 0.8 | 0.6 | 0.3 | 0.48 | -6.38 |
| 0.45 | 0.9 | 0.6 | 0.3 | 0.46 | -3.09 |
| 0.45 | 0.5 | 0.7 | 0.3 | 0.59 | -23.53 |
| 0.45 | 0.6 | 0.7 | 0.3 | 0.55 | -18.18 |
| 0.45 | 0.7 | 0.7 | 0.3 | 0.52 | -13.19 |
| 0.45 | 0.8 | 0.7 | 0.3 | 0.49 | -8.51 |
| 0.45 | 0.9 | 0.7 | 0.3 | 0.47 | -4.12 |
| 0.45 | 0.5 | 0.8 | 0.3 | 0.64 | -29.41 |
| 0.45 | 0.6 | 0.8 | 0.3 | 0.58 | -22.73 |
| 0.45 | 0.7 | 0.8 | 0.3 | 0.54 | -16.48 |
| 0.45 | 0.8 | 0.8 | 0.3 | 0.50 | -10.64 |
| 0.45 | 0.9 | 0.8 | 0.3 | 0.47 | -5.15 |
| 0.45 | 0.5 | 0.9 | 0.3 | 0.70 | -35.29 |
| 0.45 | 0.6 | 0.9 | 0.3 | 0.62 | -27.27 |
| 0.45 | 0.7 | 0.9 | 0.3 | 0.56 | -19.78 |
| 0.45 | 0.8 | 0.9 | 0.3 | 0.52 | -12.77 |
| 0.45 | 0.9 | 0.9 | 0.3 | 0.48 | -6.19 |
| 0.45 | 0.5 | 0.6 | 0.3 | 0.55 | -17.65 |
| 0.45 | 0.6 | 0.6 | 0.2 | 0.54 | -17.39 |
| 0.45 | 0.7 | 0.6 | 0.2 | 0.52 | -12.77 |
| 0.45 | 0.8 | 0.6 | 0.2 | 0.49 | -8.33 |
| 0.45 | 0.9 | 0.6 | 0.2 | 0.47 | -4.08 |
| 0.45 | 0.5 | 0.7 | 0.2 | 0.62 | -27.78 |
| 0.45 | 0.6 | 0.7 | 0.2 | 0.58 | -21.74 |
| 0.45 | 0.7 | 0.7 | 0.2 | 0.54 | -15.96 |
| 0.45 | 0.8 | 0.7 | 0.2 | 0.50 | -10.42 |
| 0.45 | 0.9 | 0.7 | 0.2 | 0.47 | -5.10 |
| 0.45 | 0.5 | 0.8 | 0.2 | 0.68 | -33.33 |
| 0.45 | 0.6 | 0.8 | 0.2 | 0.61 | -26.09 |
| 0.45 | 0.7 | 0.8 | 0.2 | 0.56 | -19.15 |
| 0.45 | 0.8 | 0.8 | 0.2 | 0.51 | -12.50 |
| 0.45 | 0.9 | 0.8 | 0.2 | 0.48 | -6.12 |
| 0.45 | 0.5 | 0.9 | 0.2 | 0.74 | -38.89 |
| 0.45 | 0.6 | 0.9 | 0.2 | 0.65 | -30.43 |
| 0.45 | 0.7 | 0.9 | 0.2 | 0.58 | -22.34 |
| 0.45 | 0.8 | 0.9 | 0.2 | 0.53 | -14.58 |
| 0.45 | 0.9 | 0.9 | 0.2 | 0.48 | -7.14 |
