# Supplementary material for: Evaluating the Hazard of Foetal Death following H1N1 Influenza Vaccination; A Population Based Cohort Study in the UK GPRD
Source: PLoS One. 2012 Dec 10;7(12):e51734. doi: 10.1371/journal.pone.0051734 (PMC3541601; doi:10.1371/journal.pone.0051734)
Supplement: Table S2 — Patient characteristics among pregnancies with and without data recorded on LMP date. (DOCX) [file pone.0051734.s002.docx]

|  | | | | | | | | | | | | |
| --- | --- | --- | --- | --- | --- | --- | --- | --- | --- | --- | --- | --- |
|  | | | **Delivery** | | | |  | | **Foetal death** | | | |
|  |  |  | **LMP available** | | **LMP defaulted** | | | **LMP available** | | | **LMP defaulted** | |
|  |  |  | **n** | **%** | **n** | **%** | | **n** | | **%** | **n** | **%** |
| **Total** | | | 16,536 | 100 | 19,902 | 100 | | 1,288 | | 100 | 2,137 | 100 |
| **Mean pregnancy length, weeks (SD)** | | | 41 | *(1.9)* | 41 | *(0.3)* | | 18 | | *(8.3)* | 13 | *(8.8)* |
| **Unvaccinated weeks** | | | . |  |  |  | |  | |  |  |  |
|  | | **weeks 1-12** | 2,198 | 3.0 | 2,699 | 2.8 | | 235 | | 32.8 | 188 | 48.5 |
|  | | **weeks 13-24** | 18,497 | 24.8 | 23,057 | 24.1 | | 314 | | 43.8 | 80 | 20.6 |
|  | | **weeks 25-43** | 53,765 | 72.2 | 69,893 | 73.1 | | 168 | | 23.4 | 120 | 30.9 |
| **Vaccinated (weeks)** | |  |  |  |  |  | |  | |  |  |  |
|  | | **weeks 1-12** | 26,799 | 9.1 | 32,626 | 8.9 | | 3,420 | | 50.5 | 3,871 | 71.6 |
|  | | **weeks 13-24** | 97,607 | 33.1 | 117,522 | 31.9 | | 3,062 | | 45.2 | 734 | 13.6 |
|  | | **weeks 25-43** | 170,180 | 57.8 | 218,012 | 59.2 | | 288 | | 4.3 | 799 | 14.8 |
| **Maternal age (years)** | | |  |  |  |  | |  | |  |  |  |
|  | **Mean (SD)** | | 30 | *(5.9)* | 30 | *(6.1)* | | 32 | | *(6.7)* | 32 | *(7.3)* |
|  | **11-19** | | 467 | 2.8 | 727 | 3.7 | | 31 | | 2.4 | 91 | 4.3 |
|  | **20-34** | | 11,395 | 68.9 | 13,955 | 70.1 | | 725 | | 56.3 | 1,185 | 55.5 |
|  | **35-40** | | 3,645 | 22.0 | 3,949 | 19.8 | | 313 | | 24.3 | 495 | 23.2 |
|  | **40-44** | | 978 | 5.9 | 1,173 | 5.9 | | 196 | | 15.2 | 311 | 14.6 |
|  | **45-49** | | 51 | 0.3 | 98 | 0.5 | | 23 | | 1.8 | 55 | 2.6 |
| **Number of previous spontaneous abortions** | | |  |  |  |  | |  | |  |  |  |
|  | **0** | | 13,689 | 82.8 | 16,400 | 82.4 | | 945 | | 73.4 | 89 | 4.2 |
|  | **1** | | 2,316 | 14.0 | 2,859 | 14.4 | | 278 | | 21.6 | 1,531 | 71.6 |
|  | **2** | | 414 | 2.5 | 515 | 2.6 | | 50 | | 3.9 | 358 | 16.8 |
|  | **>2** | | 117 | 0.7 | 128 | 0.6 | | 15 | | 1.2 | 159 | 7.4 |
| **In clinical risk group for influenza vaccination** | | |  |  |  |  | |  | |  |  |  |
|  | **No** | | 15,561 | 94.1 | 18,743 | 94.2 | | 1,205 | | 93.6 | 1,987 | 93.0 |
|  | **Yes** | | 975 | 5.9 | 1,159 | 5.8 | | 83 | | 6.4 | 150 | 7.0 |
| **Diabetes** | | |  |  |  |  | |  | |  |  |  |
|  | **No** | | 16,405 | 99.2 | 19,731 | 99.1 | | 1,272 | | 98.8 | 2,097 | 98.1 |
|  | **Yes** | | 131 | 0.8 | 171 | 0.9 | | 16 | | 1.2 | 40 | 1.9 |
| **Number of consultations in 6 months before LMP** | | |  |  |  |  | |  | |  |  |  |
|  | **0-1** | | 4,071 | 24.6 | 4,593 | 23.1 | | 304 | | 23.6 | 455 | 21.3 |
|  | **2-3** | | 3,795 | 22.9 | 4,404 | 22.1 | | 311 | | 24.1 | 443 | 20.7 |
|  | **4-5** | | 2,765 | 16.7 | 3,330 | 16.7 | | 184 | | 14.3 | 349 | 16.3 |
|  | **6-9** | | 3,256 | 19.7 | 4,002 | 20.1 | | 250 | | 19.4 | 425 | 19.9 |
|  | **10+** | | 2,649 | 16.0 | 3,573 | 18.0 | | 239 | | 18.6 | 465 | 21.8 |
| **Pre-pregnancy smoking status** | | |  |  |  |  | |  | |  |  |  |
|  | **Smoker** | | 3,797 | 23.0 | 5,176 | 26.0 | | 304 | | 23.6 | 592 | 27.7 |
|  | **Non-smoker** | | 9,142 | 55.3 | 10,609 | 53.3 | | 714 | | 55.4 | 1,078 | 50.4 |
|  | **Ex-smoker** | | 3,532 | 21.4 | 3,959 | 19.9 | | 266 | | 20.7 | 445 | 20.8 |
|  | **Unknown** | | 65 | 0.4 | 158 | 0.8 | | 4 | | 0.3 | 22 | 1.0 |
| **Pre-pregnancy BMI** | | |  |  |  |  | |  | |  |  |  |
|  | **<20** | | 1,685 | 10.2 | 2,042 | 10.3 | | 123 | | 9.5 | 211 | 9.9 |
|  | **20-24** | | 6,473 | 39.1 | 7,187 | 36.1 | | 469 | | 36.4 | 757 | 35.4 |
|  | **25-29** | | 3,367 | 20.4 | 3,790 | 19.0 | | 288 | | 22.4 | 414 | 19.4 |
|  | **30-34** |  | 1,194 | 7.2 | 1,448 | 7.3 | | 113 | | 8.8 | 148 | 6.9 |
|  | **>34** |  | 946 | 5.7 | 1,138 | 5.7 | | 85 | | 6.6 | 151 | 7.1 |
|  | **Unknown** | | 2,871 | 17.4 | 4,297 | 21.6 | | 210 | | 16.3 | 456 | 21.3 |
| **Pre-pregnancy alcohol consumption** | | |  |  |  |  | |  | |  |  |  |
|  | **Drinker** | | 10,201 | 61.7 | 11,605 | 58.3 | | 815 | | 63.3 | 1,264 | 59.1 |
|  | **Non-drinker** | | 3,709 | 22.4 | 4,141 | 20.8 | | 272 | | 21.1 | 441 | 20.6 |
|  | **Heavy drinker** | | 134 | 0.8 | 220 | 1.1 | | 16 | | 1.2 | 38 | 1.8 |
|  | **Unknown** | | 2,492 | 15.1 | 3,936 | 19.8 | | 185 | | 14.4 | 394 | 18.4 |
